# Ultrafast synthesis of near-zero-cost S-doped Ni(OH)_2_ on C_3_N_5_ under ambient conditions with enhanced photocatalytic activity†

**DOI:** 10.1039/d1ra07275g

**Published:** 2021-11-10

**Authors:** Lixiao Han, Cong Peng, Jinming Huang, Shengyao Wang, Xiaohu Zhang, Hao Chen, Yi Yang

**Affiliations:** College of Science, Huazhong Agricultural University Wuhan 430070 PR China yiyang@mail.hzau.edu.cn

## Abstract

Planting highly efficient and low-cost Ni-based noble-metal-free active sites on semiconductors is of great significance in the field of photocatalysis. Herein, taking wide visible-light-responsive 2D C_3_N_5_ as a model semiconductor, an impressive near-zero-cost 2D S-doped nickel hydroxide (S–Ni(OH)_2_) is grown on C_3_N_5_ ultrafast within 30 min under ambient conditions by facile reaction between extremely low-cost Ni(NO_3_)_2_ and Na_2_S in aqueous solution. The fabricated 2D S–Ni(OH)_2_–C_3_N_5_ hybrid exhibits enhanced photocatalytic performance for both H_2_ production from water and NO removal for air purification. The H_2_ production rate on S–Ni(OH)_2_–C_3_N_5_ is ∼7 times higher than that of Ni(OH)_2_–C_3_N_5_ and even slightly higher than that of Pt–C_3_N_5_, demonstrating its potential as a candidate for noble metal catalysts like Pt. In particular, an apparent quantum yield (AQY) value of 30.9% at 420 nm for H_2_ production is reached on 1.0 wt% S–Ni(OH)_2_–C_3_N_5_ due to quick internal charge transfer efficiency. In addition, ∼42% of NO can be purified in a continuous flow reaction system. This work affords a cost-efficient strategy to steer the photocatalytic property of Ni-based catalysts.

## Abstract 2

2D S–Ni(OH)_2_ is facially planted on 2D C_3_N_5_ at room temperature in 30 minutes *via* a reaction between Ni(NO_3_)_2_ and Na_2_S in aqueous solution. Due to quick internal charge transfer efficiency, the hybrid is highly efficient for photocatalytic H_2_ production.

## Graphical abstract

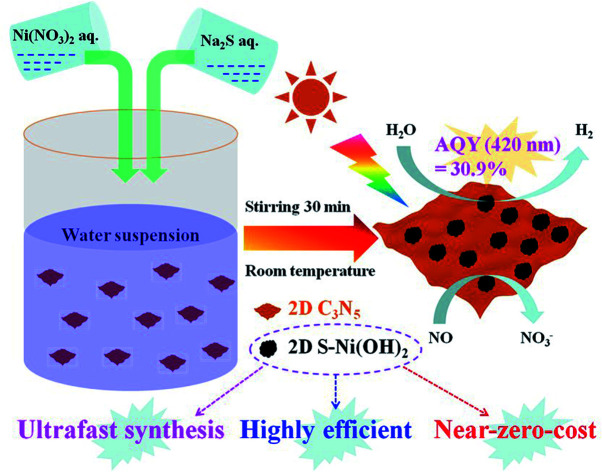

## Introduction

In the past decades, the ever-increasing energy crisis and environmental pollution have drawn wide concern considering the sustainable development of human society and public health.^1–3^ Among the various clean technologies, the environmentally friendly semiconductor-based photocatalytic oxidation and reduction is deemed as the most potential one.^4–7^ In this field, photocatalytic solar H_2_ production and NO removal lie in the core attributed to the huge energy consumption and the high risk respiratory diseases. Up to now, different semiconductors including metal oxides/sulfides (TiO_2_, CdS, ZnIn_2_S_4_, *etc.*),^8–12^ metal-free materials (carbon nitride, COFs, conjugated polymers, *etc.*)^13–17^ and heterojunctions are all widely investigated to solve these issues.^12,18–20^ Of these strategies, constructing a heterojunction between different materials to combine the superiority of each one can significantly improve the whole photocatalytic efficiency. For example, K. Domen *et al.* selectively photodeposited Rh/Cr_2_O_3_ and CoOOH on different facets of the SrTiO_3_:Al photocatalyst as cocatalysts for hydrogen and oxygen evolution respectively and an external quantum efficiency up to 96% at 350–360 nm was obtained.^21^

Recently, carbon nitride has drawn enormous attention due to its low-cost, high stability and suitable band position for water splitting;^22–25^ however, the band-gap of conventional carbon nitride (∼2.7 eV for C_3_N_4_) is still too wide to obtain ideal photocatalytic activity. To alter the photocatalytic efficiency of carbon nitride, structural and band position regulation by doping (S, O, P, N- or C-rich modification) and fabricating heterojunctions (TiO_2_/C_3_N_4_, CdS/C_3_N_4_, CNN/BDCNN, *etc.*) with type II or Z/S-scheme mechanisms are widely adopted.^15,26–31^ Recently, N-rich carbon nitride (*i.e.* C_3_N_5_) was reported;^31–36^ It possessed a narrower band-gap and higher catalytic activity than common C_3_N_4_, which represents a new direction for exploring carbon nitride based materials. For example, our previous work found that photocatalytic H_2_ production activity over Pt–C_3_N_5_ was ∼2.2 times higher than that of Pt–C_3_N_4_.^36^ Nickel based materials exhibit excellent catalytic reduction and oxidation activities in the field of both photocatalysis and electrocatalysis,^37–43^ and thus can be used as co-catalysts on C_3_N_5_. Moreover, it has been reported that S doping can significantly improve the electrocatalytic performance of Ni/Fe (oxy)hydroxide materials;^44^ thus doping of S in nickel-based co-catalysts may further improve their performance. However, these Ni-based species were commonly synthesized by hydrothermal or heat-treatment at high temperature, which was time and cost exhaustive. Therefore, construction of highly effective Ni-based catalysts in short time with low cost and energy consumption is of great importance for potential industrialization of photocatalytic technology.

**Scheme 1:**
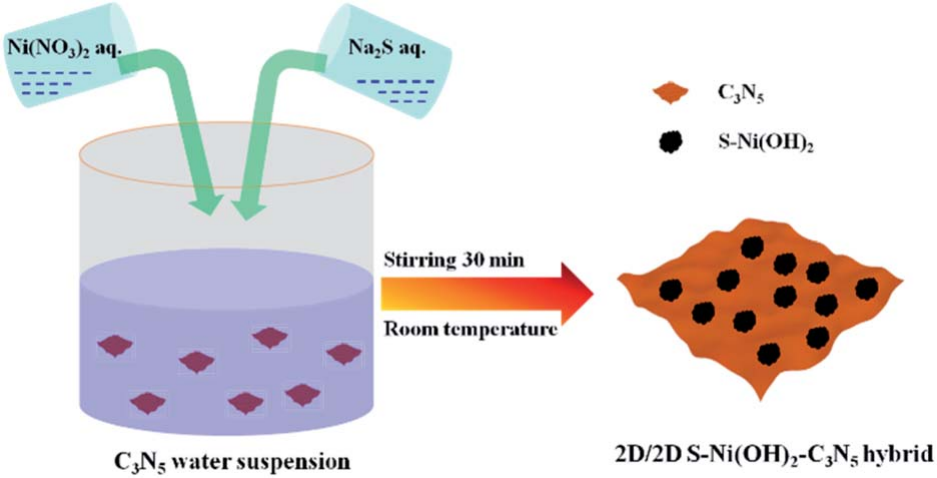
Illustration of ultrafast preparation of the S–Ni(OH)_2_–C_3_N_5_ hybrid.

Herein, taking 2D C_3_N_5_ as a model semiconductor, 2D S-doped Ni(OH)_2_ (denoted as S–Ni(OH)_2_) was facilely planted on its surface by a one-step precipitation method between Ni(NO_3_)_2_ and Na_2_S in water (co-existence of S^2−^ and OH^−^) under stirring within 30 minutes at room temperature, as revealed in Scheme 1. The hybrid photocatalyst (S–Ni(OH)_2_–C_3_N_5_) was evaluated by photocatalytic activity for H_2_ production and NO oxidation removal. Experimental results depicted that S–Ni(OH)_2_ can promote the photogenerated e^−^/h^+^ separation of C_3_N_5_ after light excitation and favor the generation of active oxygen species (ROS) participating in the subsequent photocatalytic procedure. In particular, the visible-light-induced photocatalytic H_2_ production rate over S–Ni(OH)_2_–C_3_N_5_ is even higher than that of Pt–C_3_N_5_, while the cost of S–Ni(OH)_2_ (taking the H_2_ production test as an example: in 45 mg 1.0 wt% S–Ni(OH)_2_–C_3_N_5_) is calculated to be just ∼0.0032 ¥ or ∼0.00049 $ based on the price of Ni(NO_3_)_2_ and Na_2_S. The present work provides new insights into the design of low-cost and noble-metal-free catalysts for energy and environmental applications.

## Experimental section

### Material preparation

All chemicals used were purchased from Aladdin and used without purification. C_3_N_5_ was prepared by heating 3-amino-1,2,4-triazole at 500 °C in air according to our previous work.^36^ Generally, S–Ni(OH)_2_–C_3_N_5_ was synthesized by mixing a certain amount of C_3_N_5_, Ni(NO_3_)_2_ and Na_2_S in water and stirring for 30 min. And then, the precipitate was washed with distilled water and absolute ethanol several times and dried in a vacuum at 80 °C for 12 h. The loading amount of S–Ni(OH)_2_ on C_3_N_5_ was calculated based on the initial input nickel mass fraction in the hybrid, and a series of S–Ni(OH)_2_–C_3_N_5_ hybrids with S–Ni(OH)_2_ loading amount varying between 0.5 and 3.0 wt% were prepared by changing the amount of Ni(NO_3_)_2_ and Na_2_S accordingly. Pristine S–Ni(OH)_2_ was also prepared by the same method in the absence of C_3_N_5_. By the way, Ni(OH)_2_–C_3_N_5_ was prepared by the same method using NaOH instead of Na_2_S.

### Catalyst characterization

XRD patterns were recorded on a powder X-ray diffractometer with Cu Kα radiation (D8 Advance Bruker Inc., Germany). TEM and HRTEM images were acquired on an FEI TALOS F200. SEM and EDS mapping images were acquired on a Gemini Sigma 300. The Brunauer–Emmett–Teller (BET) specific surface area was evaluated using nitrogen adsorption–desorption apparatus (ASAP 2040, Micrometrics Inc., USA) with all samples degassed at 120 °C for 12 h prior to measurements. Valence state of each element in the catalyst was analyzed by X-ray photoelectron spectroscopy (XPS) on a Thermo Fisher ESCALAB 250Xi X-ray photoelectron spectroscope. A Shimadzu UV-3100 recording spectrophotometer and fluorescence spectrometer (F-4600, Hitachi Inc., Japan) were used to record UV-vis diffuse reflectance (DRS) and PL spectra measurements respectively. Time-resolved fluorescence spectra (TRFS) were obtained on an Edinburgh FLS 1000. Electron paramagnetic resonance (EPR) spectra were recorded on a JES-FA200 EPR spectrometer. 5,5-Dimethyl-1-pyrroline-*N*-oxide (DMPO) was used as a radical spin-trapping reagent for ˙OH and ˙O_2_^−^. 2,2,6,6-Tetramethylpiperidine (TEMP) was used as the trapping agent for ^1^O_2_.^3^ The photocurrent and electrochemical impedance measurements were recorded on a CHI 660D electrochemical workstation (Chenhua Instrument, Shanghai, China) with a conventional three electrode configuration. Pt foil and Ag/AgCl (saturated KCl) were used as the counter electrode and reference electrode respectively. The working electrode was made by spreading a catalyst/Nafion slurry on FTO glass and 0.1 M Na_2_SO_4_ aqueous solution was used as the electrolyte.

### Photocatalytic activity tests and photoelectrochemical measurements

The photocatalytic H_2_ production rate and AQY measurements were carried out on a top-irradiated reaction vessel containing the catalyst and sacrificial reagent (TEOA) connected to a closed gas system with a 300 W Xe-lamp (PLS SXE300, Beijing Perfectlight Inc., China) equipped with a cutoff filter (*λ* > 420 nm) or band-pass filters (*λ* = 420, 500 nm, *etc.*); the photocatalytic H_2_ production system is shown in Fig. S1.† The H_2_ production rate was detected using a GC (SP7820, TCD detector, 5 Å molecular sieve columns, and Ar carrier), and AQY values were calculated according to our previous work:^17^1
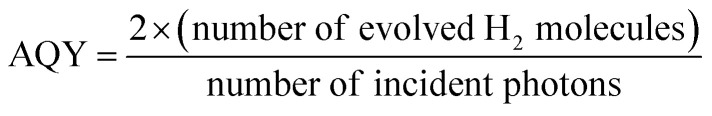


For photocatalytic NO removal, a continuous flow reactor under ambient conditions was adopted according to our previous work.^7^ The volume of the rectangular reactor was 4.5 L (30 cm × 15 cm × 10 cm). 25 mg catalyst was dispersed into the mixture of ethanol and water and then transferred into a culture dish with a diameter of 12 cm, and then the dish was placed in the reactor after the solvent was dried at 45 °C. A 30 W visible LED (General Electric) was used as a light source. The gas (containing NO and air) flow rate through the reactor was controlled at 1000 mL min^−1^ using a mass flow controller with an initial NO concentration of ∼600 ppb. The NO and NO_2_ concentrations were recorded on a NO_*x*_ analyzer model T200 (Teledyne API). The generation of NO_3_^−^ was detected by ion chromatography on a Thermo DIONEX ICS-900.

## Results and discussion

### Crystal phase and micro morphology analyses

**Fig. 1:**
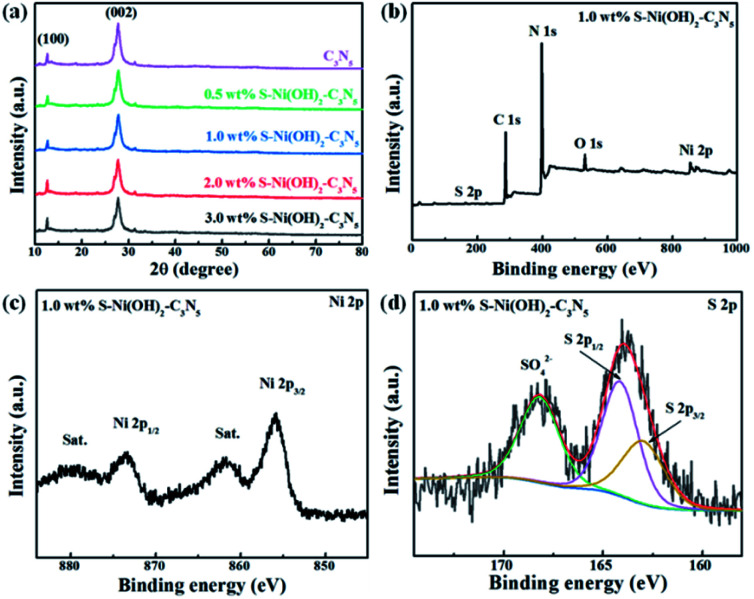
(a) XRD patterns of C_3_N_5_ and S–Ni(OH)_2_–C_3_N_5_ with different loading amounts of S–Ni(OH)_2_. (b) XPS survey, (c) Ni 2p and (d) S 2p spectra of 1.0 wt% S–Ni(OH)_2_–C_3_N_5_ respectively.

The pristine S–Ni(OH)_2_ prepared from the ultrafast reaction between Ni^2+^ and Na_2_S in water under ambient conditions within 30 min is firstly detected by XRD, as depicted in Fig. S2,† which demonstrates the formation of S–Ni(OH)_2_ with low diffraction peaks corresponding to the PDF card of 73-1520 for Ni(OH)_2_, indicating the low crystallinity of the present S–Ni(OH)_2_ material. Herein, the confirmation of S doping will be illustrated in the following section. It should be noted that pristine Ni(OH)_2_ without S doping is prepared by reaction between Ni^2+^ and NaOH for comparison, and the diffraction peaks of this Ni(OH)_2_ are also in agreement with the same PDF card (Fig. S2†), indicating that the comparison is reasonable. For the XRD patterns of C_3_N_5_ and S–Ni(OH)_2_–C_3_N_5_ hybrid (Fig. 1a), only two distinct peaks corresponding to (100) and (002) planes of C_3_N_5_,^32,35,36,45^ are observed even with a high loading amount of S–Ni(OH)_2_ up to 3.0 wt%, which should relate to the low crystallinity of present S–Ni(OH)_2_. In addition, no obvious shift happens in the main peaks of C_3_N_5_ after the loading of S–Ni(OH)_2_, revealing that the procedure for hybrid preparation does not destroy the basic structure of C_3_N_5_.

The element component of 1.0 wt% S–Ni(OH)_2_–C_3_N_5_ is analyzed by elemental analysis and ICP-MS (Table S1†). From the elemental analysis result, the C/N ratio of the catalysts was determined to be 3 : 4.7, which is much lower than that of C_3_N_4_ and very close to that of C_3_N_5_, and the result is in good agreement with previous literature.^35^ As revealed in Fig. 1b, S–Ni(OH)_2_–C_3_N_5_ is composed of C, N, Ni, O and S elements, demonstrating the existence of S-doping in Ni(OH)_2_ during the reaction between Ni^2+^ and Na_2_S preliminarily. In addition, the result depicts the successful combination of S–Ni(OH)_2_ and C_3_N_5_. The high resolution spectra of C 1s (Fig. S3a†) and N 1s (Fig. S3b†) in S–Ni(OH)_2_–C_3_N_5_ are similar to that of pristine C_3_N_5_ shown in our previous work,^36^ and no obvious peak shifts are found, revealing that the structure of C_3_N_5_ is well kept during the loading of S–Ni(OH)_2_. The existence of Ni ions is confirmed by the high resolution spectrum of Ni 2p, in which two distinct spin–orbit peaks at ∼855.8 (Ni 2p_3/2_) and ∼873.5 eV (Ni 2p_1/2_) along with two satellite peaks (identified as ‘‘Sat.’’) are clearly observed (Fig. 1c).^40,42^ The O 1s spectra mainly consist of a peak at 531.79 eV, (see Fig. S3c†) which can be assigned to the O^2−^ species in Ni(OH)_2_.^46^ Most importantly, two peaks are observed in the high resolution spectrum of S 2p, in which the peak centered at ∼164 eV represents the metal–sulfur bond and S^2−^, while the other peak in the high binding energy region can be considered as residual SO_4_^2−^ adsorbed on the material (Fig. 1d).^40,42,47^ That is, the existence of the Ni–S bond can support the formation of S-doping in Ni(OH)_2_.

**Fig. 2:**
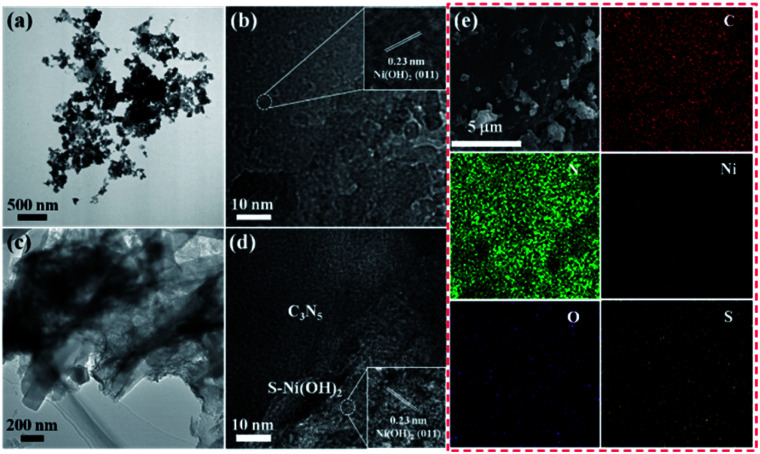
TEM and HRTEM of S–Ni(OH)_2_ (a and b) and 1.0 wt% S–Ni(OH)_2_–C_3_N_5_ (c and d). SEM and EDS mapping of 1.0 wt% S–Ni(OH)_2_–C_3_N_5_ (e).

To further confirm the successful fabrication of the S–Ni(OH)_2_–C_3_N_5_ hybrid, TEM and SEM images are acquired and shown in Fig. 2. It has been reported in our previous work that the micro morphology of pristine C_3_N_5_ is nanosheet.^36^Fig. 2a and b display the TEM and HRTEM images of S–Ni(OH)_2_. As shown in Fig. 2a, S–Ni(OH)_2_ also exhibits a micro morphology of nanosheets and a clear lattice fringe of 0.23 nm corresponding to the (011) plane of S–Ni(OH)_2_ is observed (Fig. 2b). In particular, no lattice fringe representing nickel sulfide is found, demonstrating the existence state of S-doping in S–Ni(OH)_2_. Fig. 2c displays the TEM image of 1.0 wt% S–Ni(OH)_2_–C_3_N_5_, in which 2d nanosheets containing S–Ni(OH)_2_ and C_3_N_5_ are observed. In addition, S–Ni(OH)_2_ and C_3_N_5_ come into tight contact with each other and it is difficult to distinguish each material due to their similar micro morphology. The high resolution region (Fig. 2d) of S–Ni(OH)_2_–C_3_N_5_ can be clearly divided into an amorphous region corresponding to C_3_N_5_ and a crystalline region for S–Ni(OH)_2_ with a clear lattice fringe of 0.23 nm corresponding to the (011) plane of S–Ni(OH)_2_. By the way, the two different regions in Fig. 2d come into tight contact with each other, which will be favorable for the internal transfer of photogenerated carriers.

Fig. 2e depicts the SEM and element mapping images of 1.0 wt% S–Ni(OH)_2_–C_3_N_5_. As revealed, the hybrid is basically composed of nanosheets containing S–Ni(OH)_2_ and C_3_N_5_, which is in good agreement with the above results of TEM. The element mapping images convey that the hybrid contains C, N, Ni, O and S, and all of the elements disperse uniformly in the observed region. Moreover, the existence of S-doping in S–Ni(OH)_2_ is confirmed from the mapping of S in the material. Based on the above-mentioned XRD, XPS, TEM and SEM analyses, the product of reaction between Ni^2+^ and Na_2_S is named S–Ni(OH)_2_ considering that no any information corresponding to nickel sulfide is detected while the Ni–S bond exists in the hybrid, and the hybrid of 2D S–Ni(OH)_2_–C_3_N_5_ is successfully constructed.

### DRS and e^−^/h^+^ separation analyses

**Fig. 3:**
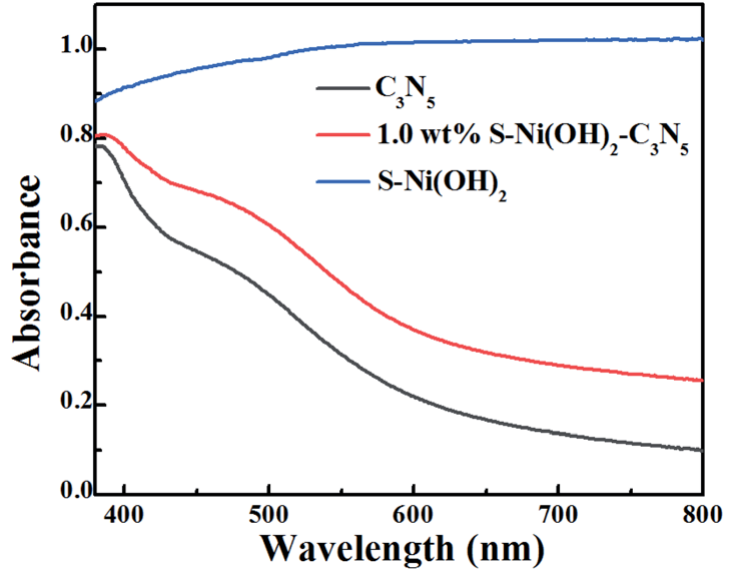
DRS spectra of S–Ni(OH)_2_, C_3_N_5_ and 1.0 wt% S–Ni(OH)_2_–C_3_N_5_.

In the field of photocatalysis, the light responsive region plays a significant role in the performance of catalysts. The visible light absorption properties of S–Ni(OH)_2_, C_3_N_5_ and S–Ni(OH)_2_–C_3_N_5_ are compared in Fig. 3. In brief, C_3_N_5_ possesses a narrow band-gap of ∼2.25 eV according to our previous work,^36^ and can absorb the visible light region of 400–600 nm with high intensity, while pristine S–Ni(OH)_2_ exhibits a strong absorption across the spectral range of 200–2400 nm (Fig. S6†). For the hybrid material, 1.0 wt% S–Ni(OH)_2_–C_3_N_5_ exhibits a similar visible light absorption region to C_3_N_5_, but the absorption intensity is slightly increased by the hybridization of black S–Ni(OH)_2_. In a word, this wide and strong visible light absorption property of the hybrid is favorable for achieving high photocatalytic performance.

**Fig. 4:**
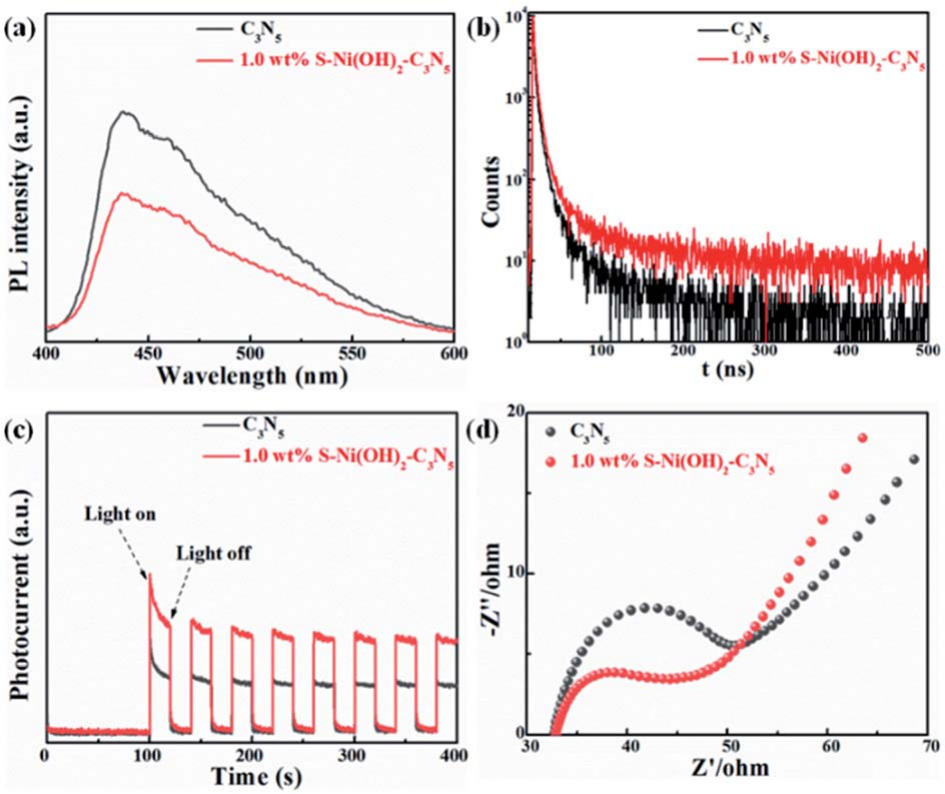
Comparison of steady-state PL (a), time-resolved PL (b), photocurrent-time (c) and EIS (d) curves of C_3_N_5_ and 1.0 wt% S–Ni(OH)_2_–C_3_N_5_.

Light absorption is the first step for a photocatalytic procedure, and the subsequent photogenerated e^−^/h^+^ separation is of great importance to the whole performance. Monitoring of the PL behavior of photocatalysts can find out some information on e^−^/h^+^ separation after light excitation.^26,48,49^ As revealed in the steady-state PL spectrum in Fig. 4a, C_3_N_5_ exhibits strong PL intensity, demonstrating its high photogenerated e^−^/h^+^ recombination rate after light excitation. However, the emission intensity of C_3_N_5_ can be efficiently quenched by S–Ni(OH)_2_. The lower PL intensity of 1.0 wt% S–Ni(OH)_2_–C_3_N_5_ than C_3_N_5_ demonstrates that the recombination of e^−^/h^+^ pairs on C_3_N_5_ after excitation is restrained, which should be caused by the electron transfer from C_3_N_5_ to S–Ni(OH)_2_ since Ni-based species such as nickel sulfide and Ni(OH)_2_ are often used as co-catalysts for photocatalytic H_2_ production.^43,50–52^ Mott–Schottky analysis was further conducted on S–Ni(OH)_2_ to verify whether the CB position of S–Ni(OH)_2_ is sufficient to capture the photogenerated electrons from C_3_N_5_ (Fig. S7†). The CB position of S–Ni(OH)_2_ was determined to be −0.58 V *vs.* NHE, which is less negative than that of C_3_N_5_ (−0.98 V *vs.* NHE).^36^ So, under irradiation, the photogenerated electrons of C_3_N_5_ could migrate to S–Ni(OH)_2_ and be captured. The time-resolved fluorescence spectra (TRFS) of C_3_N_5_ and 1.0 wt% S–Ni(OH)_2_–C_3_N_5_ are compared in Fig. 4b to further illustrate this viewpoint. As depicted, the PL intensity of C_3_N_5_ decays quickly after excitation, indicating its low fluorescence lifetime which is not favorable for photogenerated e^−^/h^+^ separation. However, the decay tendency of 1.0 wt% S–Ni(OH)_2_–C_3_N_5_ exhibits a clear slowdown under the same conditions, demonstrating that the fluorescence lifetime is prolonged, which should be related to the internal electron transfer from C_3_N_5_ to S–Ni(OH)_2_.^18,53^ For comparison, the fluorescence lifetime of C_3_N_5_ and 1.0 wt% S–Ni(OH)_2_–C_3_N_5_ is calculated to be ∼8.50 and 14.02 ns respectively *via* a biexponential fitting. The enhanced e^−^/h^+^ separation efficiency of 1.0 wt% S–Ni(OH)_2_–C_3_N_5_ compared to pristine C_3_N_5_ is further confirmed by photocurrent–time and EIS behaviors. As revealed in Fig. 4c, sharp photocurrent is generated under light irradiation and then decreases suddenly when light is turned off, demonstrating that the current is light-switched and produced by the photogenerated e^−^/h^+^ separation after the semiconductor is excited.^20,54,55^ In comparison, 1.0 wt% S–Ni(OH)_2_–C_3_N_5_ exhibits a much higher photocurrent value than pristine C_3_N_5_, indicating that the separation of photogenerated e^−^/h^+^ on C_3_N_5_ is facilitated by loading of the S–Ni(OH)_2_ co-catalyst. In addition, a smaller arc radius is recorded on the S–Ni(OH)_2_–C_3_N_5_ electrode, revealing the smaller resistance for charge transfer which is favorable for e^−^/h^+^ separation.^53,56^ All these results convey that the S–Ni(OH)_2_–C_3_N_5_ hybrid possesses quick internal charge transfer efficiency, which will facilitate the subsequent photocatalytic performance.

### Photocatalytic H_2_ production performance

**Fig. 5:**
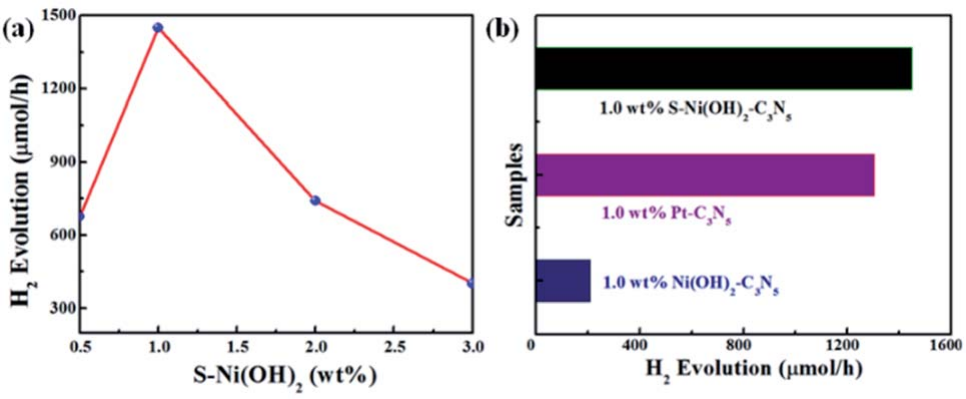
(a) Influence of S–Ni(OH)_2_ loading amount on the H_2_ production activity of the S–Ni(OH)_2_–C_3_N_5_ hybrid. (b) Comparison of H_2_ production activity over 1.0 wt% S–Ni(OH)_2_–C_3_N_5_, 1.0 wt% Ni(OH)_2_–C_3_N_5_ and 1.0 wt% Pt–C_3_N_5_ catalysts. Conditions: 45 mg catalyst, 10 vol% TEOA/water aqueous, *λ* > 420 nm.

In consideration of the wide visible light absorption region and quick photogenerated e^−^/h^+^ separation efficiency of the S–Ni(OH)_2_–C_3_N_5_ hybrid, its photocatalytic performance for H_2_ production from water with TEOA as a sacrificial reagent under visible light irradiation (*λ* > 420 nm) is evaluated firstly. A blank experiment shows that almost no H_2_ production is observed on pristine C_3_N_5_ due to the lack of a co-catalyst. And pristine S–Ni(OH)_2_ also cannot produce H_2_ under these conditions. However, as displayed in Fig. 5a, the H_2_ production rate is dramatically enhanced even with a low loading amount of 0.5 wt% S–Ni(OH)_2_ on C_3_N_5_. Generally, a volcano-type relationship between the loading amount of a cocatalyst and the whole photocatalytic activity will be obtained,^57^ suggesting that the fraction of S–Ni(OH)_2_ in the hybrid must be screened to explore the highest H_2_ production rate. As shown in Fig. 5b, the maximum H_2_ production rate is 1450 μmol h^−1^ on the catalyst of 1.0 wt% S–Ni(OH)_2_–C_3_N_5_. Notably, excessive loading of S–Ni(OH)_2_ will influence the visible light absorption of C_3_N_5_, and then the whole photoactivity is decreased. In addition, the initial concentration of Na_2_S in the procedure of catalyst preparation will influence the concentration of OH^−^ and S^2−^, which participate in the formation of S–Ni(OH)_2_. The influence of initial mol ratio between Ni^2+^ and Na_2_S (Ni/S) in the preparation procedure on the photoactivity is shown in Fig. S4,† and the selected condition for S–Ni(OH)_2_ preparation is Ni/S = 1/3.

To explore the influence of S doping on the photocatalytic H_2_ production performance, the comparison of 1.0 wt% S–Ni(OH)_2_–C_3_N_5_ and 1.0 wt% Ni(OH)_2_–C_3_N_5_ is shown in Fig. 5b. Unexpectedly, 1.0 wt% Ni(OH)_2_–C_3_N_5_ exhibits much lower H_2_ production activity (209 μmol h^−1^), which is only one seventh of the value over the 1.0 wt% S–Ni(OH)_2_–C_3_N_5_ hybrid. That is to say, a novel method is explored to prepare a highly efficient Ni(OH)_2_-based co-catalyst by simply changing the initial reactant from NaOH to Na_2_S that reacts with Ni^2+^ under ambient conditions ultra-fast. The photocatalytic performance is also evaluated using Pt nanoparticles as a reference since Pt is generally considered as the most efficient co-catalyst for photocatalytic H_2_ production.^15,23^ As depicted in Fig. 5b, the H_2_ production rate on 1.0 wt% S–Ni(OH)_2_–C_3_N_5_ is slightly higher than that of 1.0 wt% Pt–C_3_N_5_, demonstrating that the present S–Ni(OH)_2_ is a highly efficient co-catalyst for H_2_ production and is promising in consideration of the facile preparation procedure and low cost of Ni-based materials compared with noble metals like Pt.

**Fig. 6:**
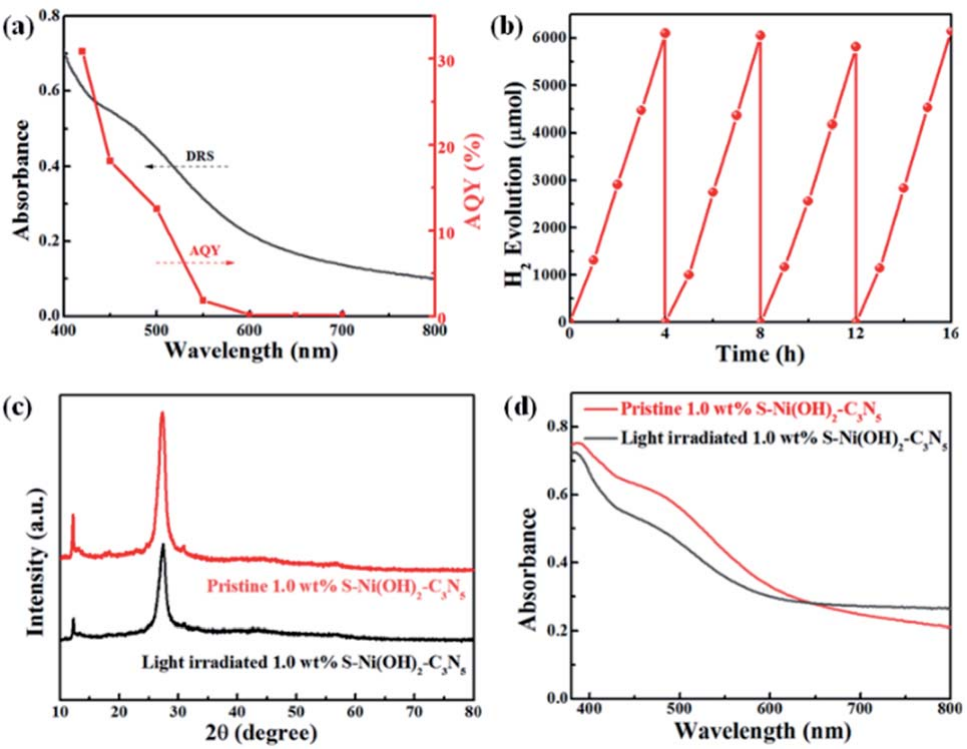
(a) Changing tendency of AQY values of 1.0 wt% S–Ni(OH)_2_–C_3_N_5_ with monochromatic light irradiation. (b) Cycle performance of 1.0 wt% S–Ni(OH)_2_–C_3_N_5_ for H_2_ production under visible light irradiation. Comparison of XRD (c) and DRS (d) spectra of 1.0 wt% S–Ni(OH)_2_–C_3_N_5_ before and after photocatalytic performance.

The AQY values of 1.0 wt% S–Ni(OH)_2_–C_3_N_5_ under a series of monochromatic light irradiation are shown in Fig. 6a, in which the AQY values decrease gradually with the increase of light wavelength and the highest AQY value (30.9%) is obtained at 420 nm. The change tendency of AQY is in agreement with the DRS spectrum of S–Ni(OH)_2_–C_3_N_5_, demonstrating that the photocatalytic performance of S–Ni(OH)_2_–C_3_N_5_ is determined by its light absorption behavior. Long-term stability of a photocatalyst is an important norm in the field of H_2_ production. Fig. 6b reveals the cycling performance of 1.0 wt% S–Ni(OH)_2_–C_3_N_5_ and no obvious attenuation of H_2_ production rate happens after 16 h and 4 runs of photocatalytic test, demonstrating the excellent photocatalytic stability of the present S–Ni(OH)_2_–C_3_N_5_ hybrid. To further evaluate the stability of S–Ni(OH)_2_–C_3_N_5_ in photocatalysis, the samples are collected after long-term light irradiation and monitored by XRD and DRS. As depicted in Fig. 6c and d, no obvious change happens in the XRD peaks of S–Ni(OH)_2_–C_3_N_5_ experiencing the photocatalytic test, indicating that the basic structure of the hybrid is maintained. Meanwhile, the collected sample exhibits a similar visible light absorption region compared with fresh S–Ni(OH)_2_–C_3_N_5_, demonstrating the stability of the hybrid again.

### Photocatalytic NO oxidation performance

**Fig. 7:**
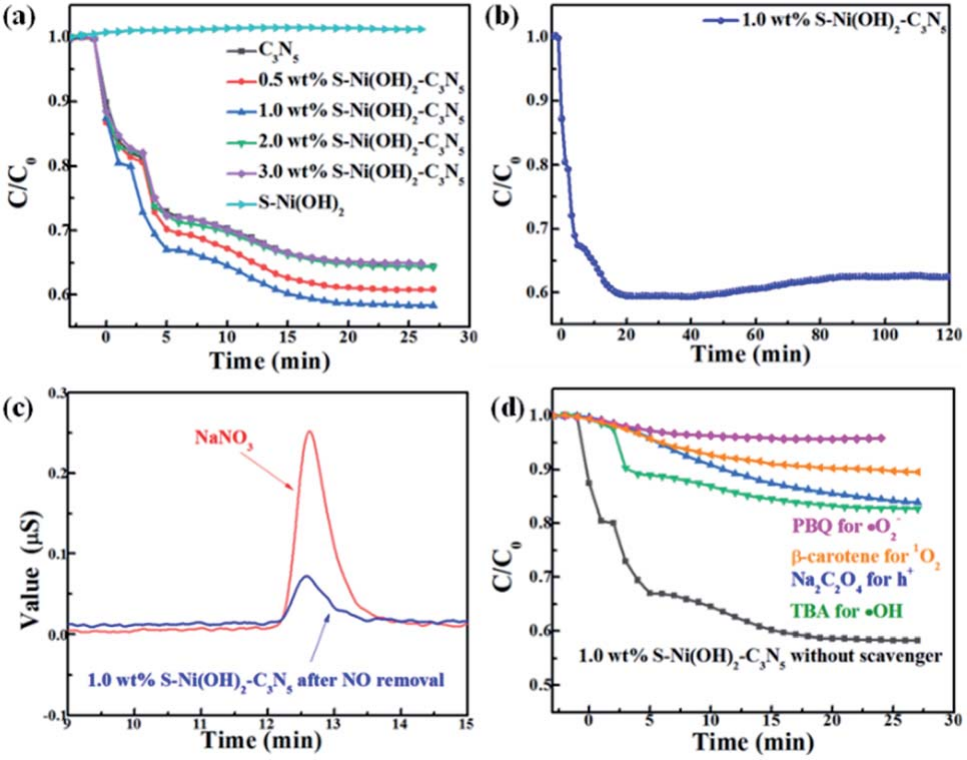
(a) Influence of S–Ni(OH)_2_ loading amount on NO removal ratio over the S–Ni(OH)_2_–C_3_N_5_ hybrid. (b) Durability of 1.0 wt% S–Ni(OH)_2_–C_3_N_5_ for NO removal. (c) Detection of NO_3_^−^ by ion chromatography. (d) Photocatalytic NO removal performance of 1.0 wt% S–Ni(OH)_2_–C_3_N_5_ in the presence of a series of trapping agents.

Except for H_2_ production, the photocatalytic performance of S–Ni(OH)_2_–C_3_N_5_ is also tested for NO oxidation to demonstrate the potential of the catalyst in purification of atmospheric pollution. A control experiment shows that pristine S–Ni(OH)_2_ has no activity for NO removal. As revealed in Fig. 7a, the oxidation of NO can reach equilibrium in 25 min in the present continuous flow reaction system. Briefly, NO removal efficiencies on C_3_N_5_ and S–Ni(OH)_2_–C_3_N_5_ with 0.5, 1.0, 2.0, and 3.0 wt% S–Ni(OH)_2_ fraction after 25 min of light irradiation are 35.0, 37.0, 42.0, 35.0 and 35.0% respectively and the most efficient catalyst is 1.0 wt% S–Ni(OH)_2_–C_3_N_5_ hybrid, indicating that the photocatalytic performance of C_3_N_5_ is enhanced by loading of S–Ni(OH)_2_. And the above-mentioned quick internal charge transfer from C_3_N_5_ to S–Ni(OH)_2_ under light excitation should play a significant role in this process. Fig. 7b evaluates the durability and stability of the 1.0 wt% S–Ni(OH)_2_–C_3_N_5_ hybrid for NO removal, and only a slight decrease in NO removal ratio is observed during 120 nm light irradiation, demonstrating the good stability of the hybrid. Generally, the main products of photocatalytic NO oxidation are NO_2_ and NO_3_^−^ according to literature reports.^58–60^ The detection of NO_2_ is carried out on the present NO_*x*_ analyzer, and the concentration of NO_2_ along with NO oxidation is shown in Fig. S5.† It is found that most NO is converted to NO_3_^−^ based on the amount of NO removal and NO_2_ production. Therefore, the confirmation of produced NO_3_^−^ in the reaction is shown in Fig. 7c with NaNO_3_ as a reference by ion chromatography, in which a clear signal corresponding to NO_3_^−^ is observed for the sample prepared with 1.0 wt% S–Ni(OH)_2_–C_3_N_5_ experiencing photocatalytic NO oxidation reaction.

**Fig. 8:**
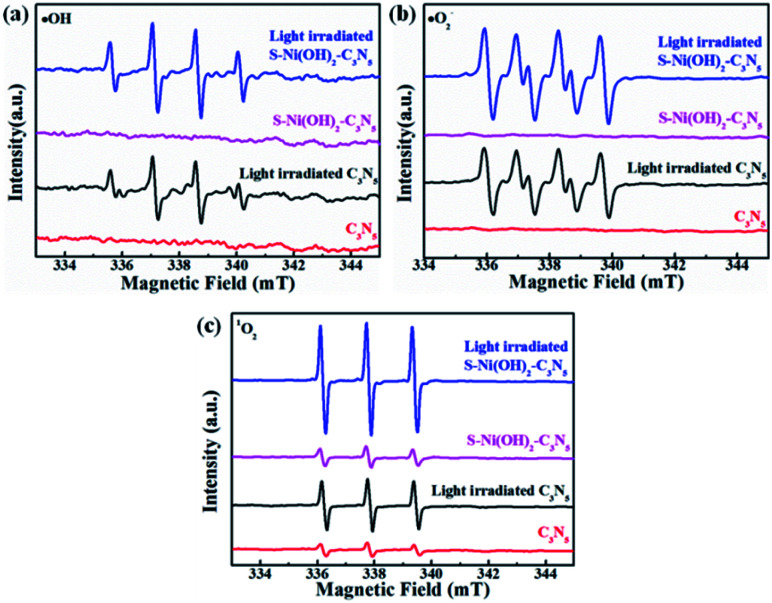
ESR spectra of DMPO-˙OH (a), DMPO-˙O_2_^−^ (b) and TEMPO (c) for ROS detection.

It has been reported that activation of molecular O_2_ and generation of a series of ROS (*i.e.* ˙OH, ˙O_2_^−^ and ^1^O_2_) play the main role in the procedure of photocatalytic NO oxidation.^61–63^ In addition, photogenerated h^+^ residual in the VB of the semiconductor is also of great importance.^64,65^ Thus, the photocatalytic NO removal performance of 1.0 wt% S–Ni(OH)_2_–C_3_N_5_ in the presence of a series of trapping agents for different active species is compared in Fig. 7d. As displayed, when Na_2_C_2_O_4_, *tert*-butanol (TBA), *p*-benzoquinone (PBQ) and β-carotene are used as scavengers for h^+^, ˙OH, ˙O_2_^−^ and ^1^O_2_ respectively, the photocatalytic performance of 1.0 wt% S–Ni(OH)_2_–C_3_N_5_ is seriously restrained, demonstrating that NO is removed by these ROS and residual h^+^ in the VB of C_3_N_5_. The NO_2_ concentration during the photocatalytic NO removal on 1.0 wt% S–Ni(OH)_2_–C_3_N_5_ was also monitored with the NO_*x*_ analyzer (see Fig. S8†). The corresponding residual ratio of NO_2_ is provided in Table S2.† From these results, it can be seen that the ˙OH and ^1^O_2_ play important roles in the conversion of NO_2_ to NO_3_^−^. Electron spin-resonance spectroscopy (ESR) tests are conducted to monitor the change of signals corresponding to ˙OH, ˙O_2_^−^ and ^1^O_2_. As revealed in Fig. 8, almost no signals are detected in the dark on both 1.0 wt% S–Ni(OH)_2_–C_3_N_5_ and C_3_N_5_, but strong peaks corresponding to distinct DMPO-˙OH, DMPO-˙O_2_^−^ and TEMPO (product of TEMP oxidized by ^1^O_2_) signals are detected,^3,7,61^ demonstrating that the generation of these ROS is light controllable. Moreover, the light induced ESR signal intensity on 1.0 wt% S–Ni(OH)_2_–C_3_N_5_ is higher than that of C_3_N_5_, revealing that more amount of ROS is generated on S–Ni(OH)_2_–C_3_N_5_ due to the quick internal charge transfer. So, the process of photocatalytic NO removal on 1.0 wt% S–Ni(OH)_2_–C_3_N_5_ should be as follows:

2 S–Ni(OH)_2_–C_3_N_5_ + *hv* → e^−^ + h^+^

3 e^−^ + h^+^ → ˙O_2_^−^

4 h^+^ + ˙O_2_^−^ → ^1^O_2_

5 ˙O_2_^−^ + H_2_O → ˙OH

6 ˙O_2_^−^/^1^O_2_/h^+^/˙OH + NO → NO_2_

7 ^1^O_2_/˙OH + NO_2_ → NO_3_^−^

**Fig. 9:**
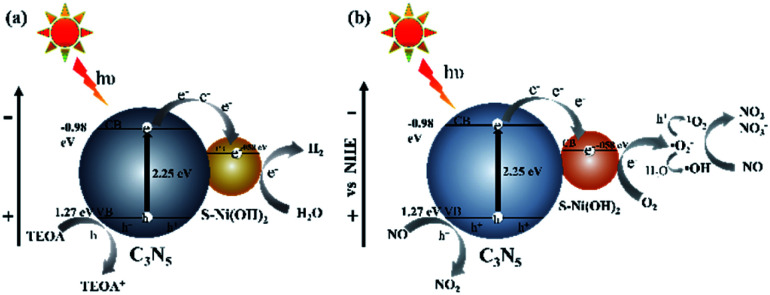
The supposed mechanism of photocatalytic H_2_ production (a) and NO removal (b) on 1.0 wt% S–Ni(OH)_2_–C_3_N_5_.

Based on all of the above results, the mechanism for photocatalytic H_2_ production or NO oxidation over the S–Ni(OH)_2_–C_3_N_5_ hybrid is proposed. Under visible light irradiation, C_3_N_5_ is excited and generates e^−^/h^+^ pairs. S–Ni(OH)_2_ can promote the internal charge separation and harvest the e^−^ on the CB of C_3_N_5_, and then the trapped electrons participate in subsequent surface reactions. For photocatalytic H_2_ production (Fig. 9a), the collected electrons on S–Ni(OH)_2_ react with adsorbed H_2_O molecules and H_2_ is produced. The residual h^+^ on the VB of C_3_N_5_ is consumed by TEOA, and then the whole photocatalytic H_2_ production procedure is accomplished. However, the situation for photocatalytic NO oxidation is different (Fig. 9b). O_2_ is firstly activated and a series of ROS (˙O_2_^−^, ^1^O_2_ and ˙OH) can be generated. Generally, ˙O_2_^−^ is produced by reaction between e^−^ and adsorbed O_2_, while the reaction between ˙O_2_^−^ and h^+^ can generate ^1^O_2_.^3,61^ ˙OH should originate from the oxidization of H_2_O by ˙O_2_^−^ since the VB position of C_3_N_5_ cannot satisfy the requirement of ˙OH generation based on our previous work.^33,66^ In addition, the generation of these ROS is enhanced by S–Ni(OH)_2_, as discussed in the above section. And then, these ROS and the residual h^+^ on the VB of C_3_N_5_ participate in the NO oxidation procedure along with the formation of NO_3_^−^ and NO_2_.

## Conclusions

In summary, near-zero-cost 2D S–Ni(OH)_2_ active sites are planted on wide visible light responsive C_3_N_5_ ultrafast within 30 min at room temperature by reaction between Ni(NO_3_)_2_ and Na_2_S in aqueous solution. The loading of S–Ni(OH)_2_ can greatly enhance the photogenerated e^−^/h^+^ separation efficiency of C_3_N_5_ after light excitation. Due to the quick internal charge transfer, the S–Ni(OH)_2_–C_3_N_5_ hybrid is highly efficient as a multifunctional catalyst in various photocatalytic applications: H_2_ production from water and NO removal. Most impressively, the H_2_ production activity on S–Ni(OH)_2_–C_3_N_5_ is even higher than that of Pt–C_3_N_5_ and an AQY value of 30.9% at 420 nm is achieved. This work brings new insights into the design of low-cost noble-metal-free co-catalysts on semiconductors for photocatalytic applications.

## Conflicts of interest

There are no conflicts to declare.

## Supplementary Material

RA-011-D1RA07275G-s001
