# Supplementary material for: Ultrafast synthesis of near-zero-cost S-doped Ni(OH)2 on C3N5 under ambient conditions with enhanced photocatalytic activity
Source: RSC Adv. 2021 Nov 10;11(57):36166–73. doi: 10.1039/d1ra07275g (PMC9043441; doi:10.1039/d1ra07275g)
Supplement: RA-011-D1RA07275G-s001 [file RA-011-D1RA07275G-s001.pdf]

Electronic Supplementary Material (ESI) for RSC Advances.
This journal is © The Royal Society of Chemistry 2021

Ultrafast synthesis of near-zero-cost S-doped $Ni(OH)_2$ on C_3N_5 at ambient condition with enhanced photocatalytic activity

Lixiao Han#, Cong Peng#, Jinming Huang, Shengyao Wang, Xiaohu Zhang, Hao Chen, Yi Yang*

College of Science, Huazhong Agricultural University, Wuhan 430070, PR China

yiyang@mail.hzau.edu.cn

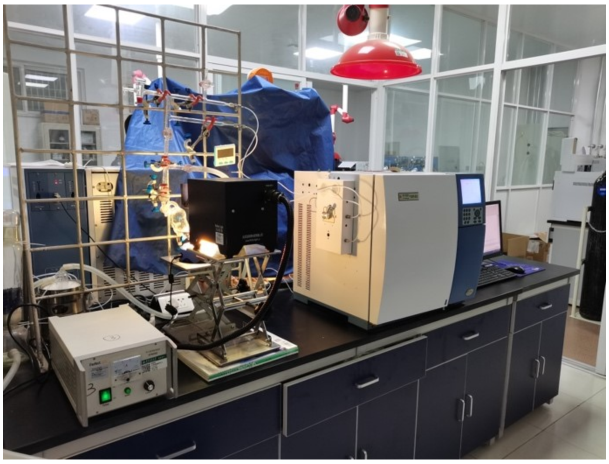

Fig. S1. The photograph of photocatalytic H_2 system.

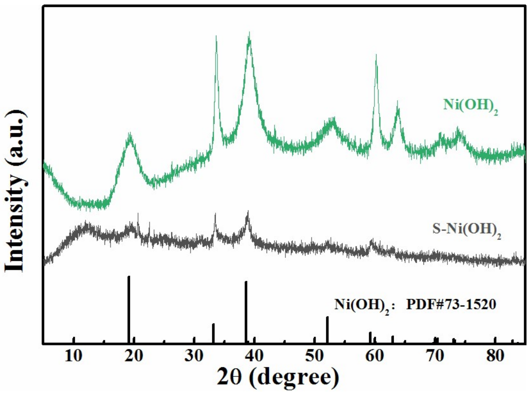

Fig. S2. XRD patterns of S-$Ni(OH)_2$ and $Ni(OH)_2$.

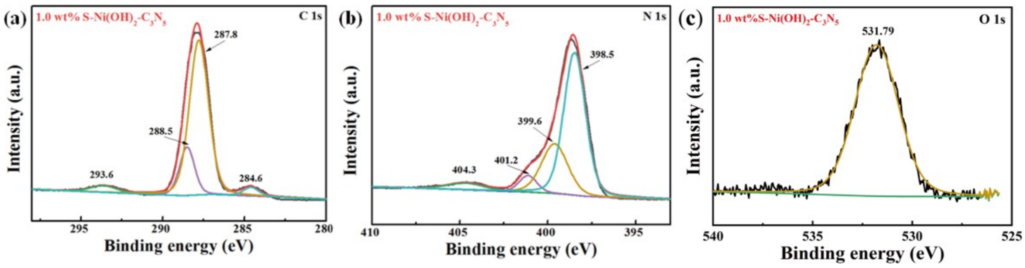

Fig. S3. High-resolution XPS spectra of C 1s (a), N 1s (b), and O 1s (c) of 1.0 wt% S-Ni(OH)$_2$-C$_3$N$_5$.

Table S1. The element content of C, N, S, (determined by elemental analysis) and Ni (determined by ICP-MS) of the prepared S-Ni(OH)$_2$-C$_3$N$_5$ materials.

Samples	N (wt%)	C (wt%)	S (wt%)	Ni (wt%)
0.5 wt% S-Ni(OH)$_2$-C$_3$N$_5$	59.38	32.91	0.341	0.3
1.0 wt% S-Ni(OH)$_2$-C$_3$N$_5$	57.64	31.42	0.555	0.98
2.0 wt% S-Ni(OH)$_2$-C$_3$N$_5$	56.5	31.26	1.509	1.6

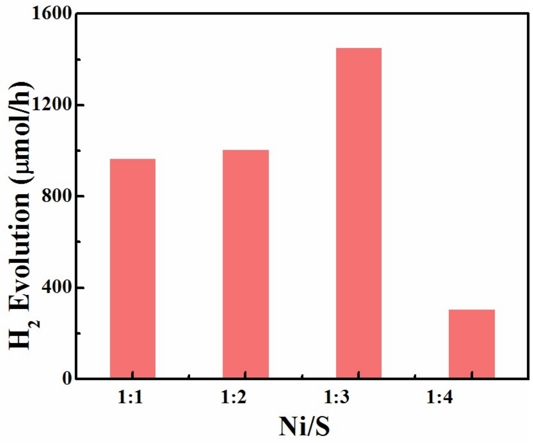

Fig. S4. Influence of mol ratio of Ni^{2+}/Na$_2$S on the H$_2$ production activity of 1.0 wt% S-Ni(OH)$_2$-Ni(OH)$_2$.

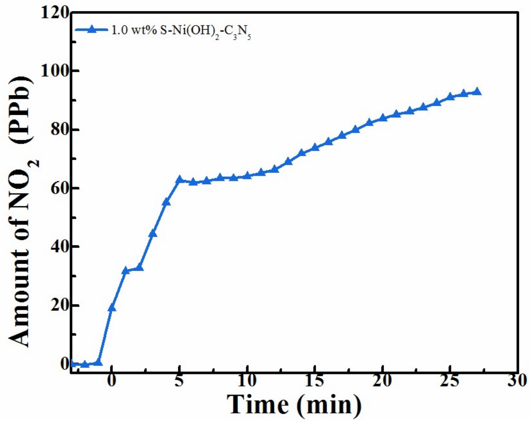

Fig. S5. Change tendency of NO$_2$ during NO oxidation procedure over 1.0 wt% S-Ni(OH)$_2$-C$_3$N$_5$.

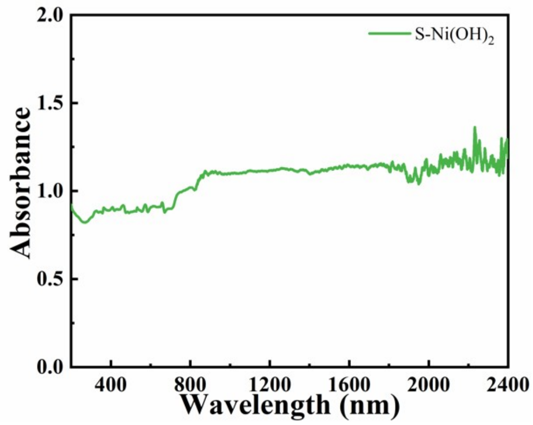

Fig. S6. The solid-state UV-Vis-NIR diffuse reflectance spectra (DRS) of S-Ni$(OH)_2$.

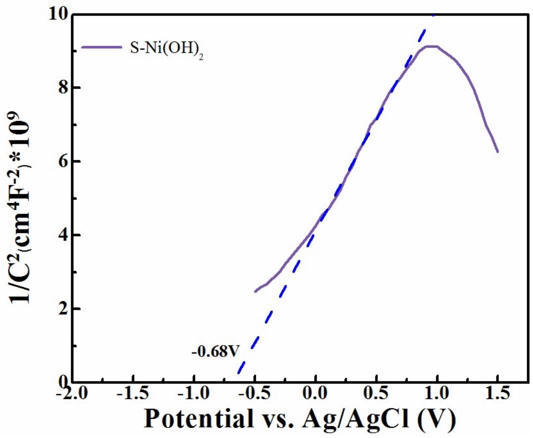

Fig. S7. The Mott - Schottky plots of S-Ni$(OH)_2$.

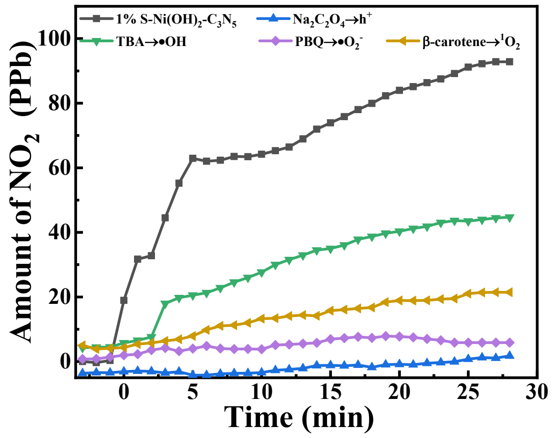

Fig. S8. The NO_2 concentration during the photocatalytic NO removal with scavengers on 1.0 wt% S-Ni$(OH)_2$-C_3N_5.

Table S2. The residual ratio of NO_2 by using different scavengers on 1.0 wt% S-Ni(OH)$_2$-C_3N_5.

*Scavenger	No scavenger	$Na_2C_2O_4$ (h^+)	TBA (•OH)	PBQ (•O_2^-)	β-carotene ($^1O_2$)
**Residual ration of NO_2	26.4%	2.0%	**40.7%**	20.0%	**35.0%**

* $Na_2C_2O_4$ for h^+, TBA for •OH, PBQ for •O_2^- and β-carotene for $^1O_2$

**Residual ration of $NO_2=C_{NO2}/\Delta C_{NO}$
